# Goblet cell adenocarcinoma of the anal canal with perianal Paget disease: A rare case report with literature review

**DOI:** 10.1097/MD.0000000000033598

**Published:** 2023-04-21

**Authors:** Minhua Li, Xiaofei Yao

**Affiliations:** a Department of Pathology, Shaoxing People’s Hospital, Shaoxing, Zhejiang, China.

**Keywords:** anal canal, carcinoids, goblet cell adenocarcinoma, mixed adenoneuroendocrine carcinoma, neuroendocrine carcinoma, Paget disease

## Abstract

AS an uncommon neoplasm, goblet cell adenocarcinoma (GCA) is characterized by mixed endocrine-exocrine features. It is almost exclusively found in the appendix. Primary GCA of the anal canal is extremely rare.

**Case presentation:**

Herein we describe a novel rare case of 74-year-old Chinese female who is diagnosed with GCA in the anal canal with perianal Paget disease, including a brief review of the literature. In the lesion of anal canal, the tumor was composed of signet-ring-like cells on confluent growth model and copious mucin was produced as well. Simultaneously, the results of immunohistochemistry showed signet-ring-like cells were positive for CK20, CDX2, synaptophysin (Syn), CD56, carcinoembryonic antigen (CEA) and Villin. Meanwhile, the Ki67-labeling index reached 40%. In the lesion of perianal Paget disease, the small groups of atypical neoplastic cells were present in the epidermis. Immunohistochemically, the neoplastic cells were positive for CK20, CDX2 and epithelial membrane antigen, but negative for CK7, GCDFP15, S100, HMB45, and P63. The Ki67-labeling index reached 60% in the most concentrated spot.

**Conclusions:**

Extra-appendiceal GCA was rare and easily under-recognizable. The diagnosis of GCA was seldom made preoperatively. Occasionally, GCA could occur in the anal canal accompanied by perianal Paget disease. So careful rectal examination was important in the patient with perianal Paget disease for avoid missing diagnosis of GCA on anal canal. GCA may show aggressive clinical behavior compared with typical well-differentiated neuroendocrine tumors. Therefore, we should pay more attention on the recognization of this rare disease.

## 1. Introduction

Goblet cell adenocarcinoma (GCA) is predominantly composed of goblet cells. Occasionally, neuroendocrine cells and Paneth cells are also present. The cells often arrange in small tubules, which is similar to intestinal crypts. Typically, the tumor cells show very little cytologic atypia and mitotic figures are rare as well. Both neuroendocrine granules and mucin droplets are present in the same cells, meanwhile, the tumor cells are positive for epithelial markers and neuroendocrine markers, resulting in the origin of cell has historically been elusive. So the nomenclature and classification of GCA have been the subject of debate for a long time. In 1969, the authors first described these tumors, pointing out that they represented a class of tumors with features intermediate between adenocarcinomas and digestive tract carcinoids.^[1]^ Subsequently, many different names were introduced for these tumors, including Mucinous carcinoid tumor, goblet cell carcinoid, well-differentiated adenocarcinoma adenocarcinoid, crypt cell carcinoma, adenocarcinoma ex-goblet cell carcinoid, and mixed adenoneuroendocrine carcinoma.^[2]^ Until recently, the WHO classificatory system claimed that these tumors should not be considered as a variant of neuroendocrine tumors. They were more appropriately grouped with other adenocarcinomas in terms of their behavior and biology. In 2019, GCA was formally recommended by World Health Organization.

Appendix is the most common site for GCA though extra-appendix sites, such as the duodenum, bronchus, stomach, biliary tract, small intestine and colon, have also been reported.^[1,3–6]^ Compared to appendiceal GCA, GCA in the lower gastrointestinal tract is exceedingly rare. There are only rare cases was reported about the GCA of rectum and its etiology is still unclear.^[4,7–14]^ Our study gave a detailed account of the first case of anal GCA accompanied with perianal Paget disease encountered in a 74-year-old female.

## 2. Case presentation

A 74-year-old Chinese woman consulted her local physician about she has a history of perianal pruritus and hematochezia for nearly a month. So she was admitted to our hospital for investigations and treatment. At physical examination, eczematous plaque with white scaling and ulcerations was found on the left side of the anus, which measured about 5 × 3 cm. On digital rectal examination, a hard tumor was palpable on the left anterior wall of the anal canal, which measured about 1 cm in radius. Prior to this presentation, she did not have history of acute appendicitis. No lymph node metastasis or remote metastatic lesions were found on the computed tomography scan. Laboratory tests showed almost all of the tumor markers were normal and colonoscopic examination did not show any abnormalities. Subsequently, histological examination of biopsies was performed on the perianal skin and anal canal. The diagnosis of GCA in the anal canal with perianal Paget disease was made. About a week later, Miles’ operation with lymph node dissection was performed. After the surgery, several chemotherapy regimens were given and the patient was stable for 24 months.

The tissue was fixed in formalin, embedded in paraffin and 4 um thin sections were cut and stained with hematoxylin and eosin. Immunohistochemical staining was performed using commercially available antibodies to the following antigens: synaptophysin (Syn), CD56, chromogranin A, CDX2, carcinoembryonic antigen (CEA), Villin, CK 20, epithelial membrane antigen, CK7, GCDFP-15, S100, HMB45, P63, and Ki-67. All the protocols were employed according to the manufacturers’ recommendations (Tables 1 and 2).

**Table 1:** Summary of primary antibodies and results of immunohistochemistry in GCA.

Antibody	Source	Dilution	Result
SyN	Ascend bio, Guangzhou, China	1:100	+
CD56	Ascend bio, Guangzhou, China	1:100	+
CgA	Ascend bio, Guangzhou, China	1:100	−
CDX2	Ascend bio, Guangzhou, China	1:400	+
CEA	Ascend bio, Guangzhou, China	1:200	+
Villin	Ascend bio, Guangzhou, China	1:400	+
Ki-67	Ascend bio, Guangzhou, China	1:200	40% in the most concentrated spot
CK20	Ascend bio, Guangzhou, China	1:200	+
CK7	Ascend bio, Guangzhou, China	1:100	−

CEA = carcinoembryonic antigen, CgA = chromogranin A, CK = cytokeratin, GCA = goblet cell adenocarcinoma, SyN = synaptophysin;

**Table 2:** Summary of primary antibodies and results of immunohistochemistry in Paget disease.

Antibody	Source	Dilution	Result
EMA	Ascend bio, Guangzhou, China	1:100	+
CK7	Ascend bio, Guangzhou, China	1:100	−
GCDFP-15	Ascend bio, Guangzhou, China	1:200	−
HMB45	Ascend bio, Guangzhou, China	1:400	−
Ki-67	Ascend bio, Guangzhou, China	1:200	60% in the most concentrated spot
P63	ZSGB bio, Beijing, China	1:200	−
S-100	Dako, Glostrup, Denmark	1:3200	−
CK20	Ascend bio, Guangzhou, China	1:200	+
CDX2	Ascend bio, Guangzhou, China	1:400	+

CK = cytokeratin, EMA = epithelial membrane antigen.

**Figure 1.**
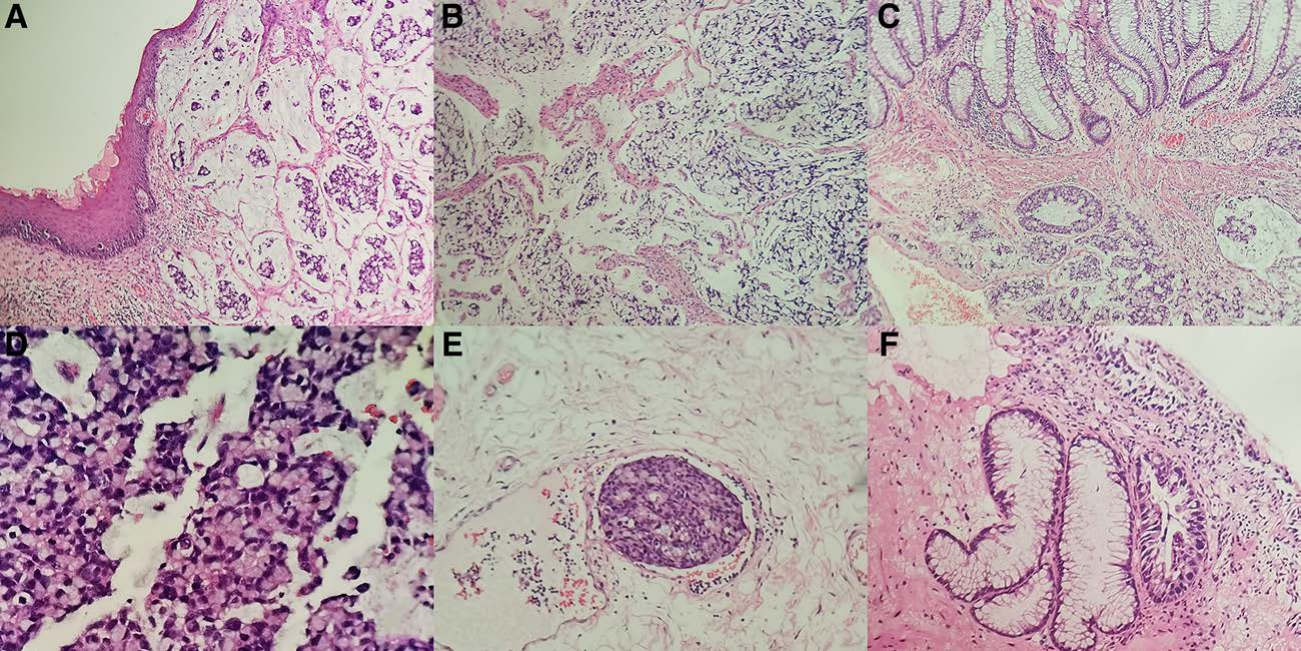
(A) On the background of copious mucin, the tumor lesion was revealed in submucosal layer of the anal canal (×200). (B) The obvious muscular invasion was showed (×200). (C) The submucosal layer of lower rectum was invaded by the tumor (×200). (D) The tumor was composed of signet-ring-like cells on confluent growth model (×400). (E) Focally, the vessel invasion was found (×200). (F) Peripheral to the normal anal glands, the individual adenocarcinoma in situ was displayed (×200).

**Figure 2.**
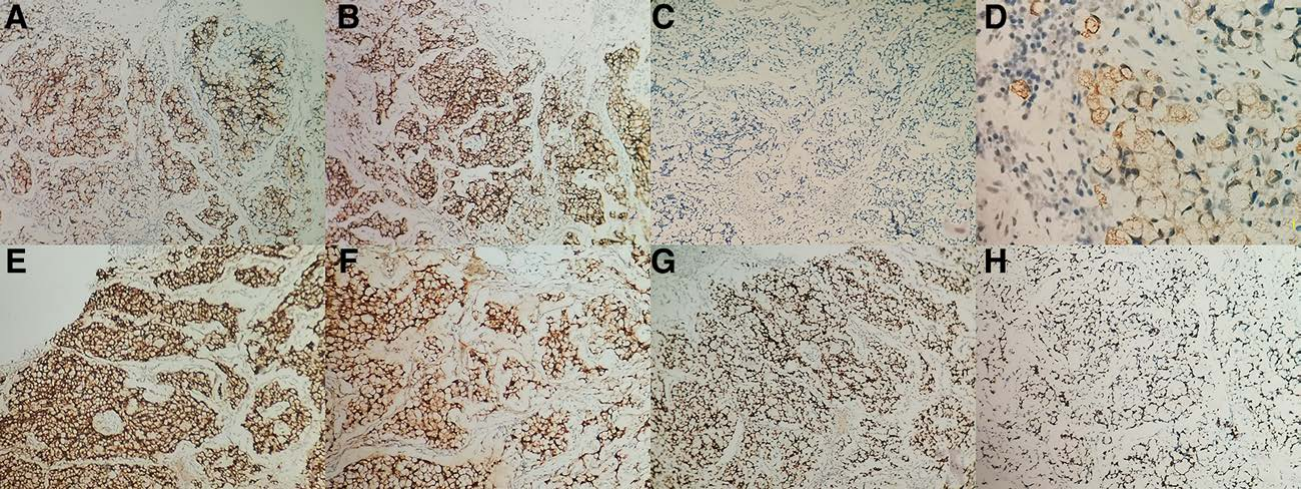
(A) Positive staining of Syn was displayed (×200). (B) Strong staining of CD56 was showed (×200). (C) Negative staining for CgA was shown (×200). (D) Positivity of CK20 was evident (×400). (E) Strong staining of CEA was revealed (×200). (F) Positive staining of Villin was displayed (×200). (G) Nuclear staining for CDX2 was shown (×200). (H) The Ki67-labeling index reached 40% in the most concentrated spot (×200). CgA = chromogranin A, SyN = synaptophysin.

Histological examination of the anal canal revealed an invasive lesion in submucosal and muscular layer, which mainly in the anal canal but focally invading the lower rectum (Fig. 1A–C). The tumor was composed of signet-ring-like cells on confluent growth model and copious mucin was produced as well (Fig. 1D). Focally, the vessel invasion was found as well (Fig. 1E). Peripheral to the normal anal glands, we found individual adenocarcinoma in situ (Fig. 1F). Unfortunately, the transition between the confluent nest of signet-ring-like cells and Paget disease in the epidermis was not found. Simultaneously, the results of immunohistochemistry showed signet-ring-like cells were positive for Syn, CD56, CDX2, CEA, Villin, and cytokeratin (CK) 20 (Fig. 2A, B, D–G). However, Negative staining for chromogranin A was shown (Fig. 2C). The Ki67-labeling index reached 40% in the most concentrated spot (Fig. 2H). Therefore, we made a diagnosis of GCA in this patient.

**Figure 3.**
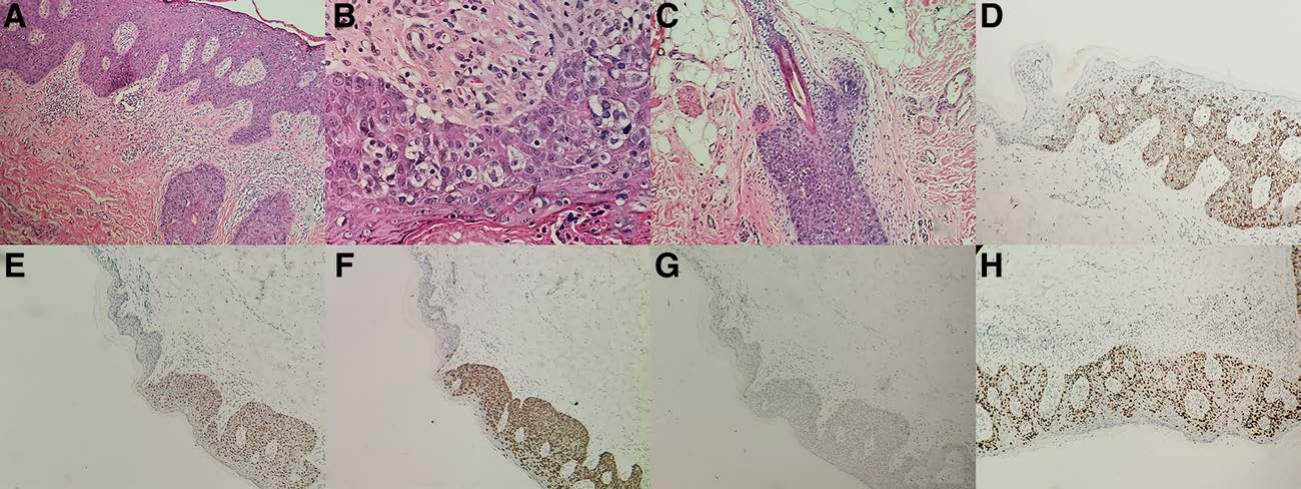
(A) The isolated/small groups of atypical neoplastic cells were present in the epidermis (×200). (B) The cells showed pale clear cytoplasm and large hyperchromatic nuclei (×400). (C) The hair follicle was involved by the tumor cells (×200). (D) Positive staining of EMA was displayed (×200). (E) Nuclear staining for CDX2 was shown (×100). (F) Positivity of CK20 was evident (×100). (G) Negative staining for GCDFP-15 was shown (×100). (H)The Ki67-labeling index reached 60% in the most concentrated spot (×200). EMA = epithelial membrane antigen.

Meanwhile, on the lesion of perianal Paget disease, the result showed isolated/small groups of atypical neoplastic cells were present in the epidermis without dermal invasion (Fig. 3A). The cells showed pale clear cytoplasm and large hyperchromatic nuclei (Fig. 3B). Focally, the hair follicle was involved by the tumor cells (Fig. 3C). Immunohistochemically, the neoplastic cells were positive for CK20, CDX2 and epithelial membrane antigen (Fig. 3D–F) but negative for CK7, GCDFP15, S100, HMB45, and P63 (Fig. 3G). The Ki67-labeling index reached 60% in the most concentrated spot (Fig. 3H). Therefore, we made a diagnosis of Paget disease in this patient.

## 3. Discussion

The gastrointestinal epithelium consist of various different kinds of cells, each kind of cells carries out specialized functions. Among these cells, there is Paneth cells that release antimicrobial factors, entero-endocrine cells that play a important role in secreting hormones and goblet cells which are responsible for secrete mucins.^[1]^ Beside barrier maintenance function of the mucins, the goblet cells also secrete chemokines, cytokines and anti-microbial proteins which play a key role in innate immunity.^[15]^ As far as we all know, goblet cells was usually found in the reproductive system, conjunctiva, respiratory tract and gastrointestinal tracts, and other columnar epithelial cells often surround them.^[15,16]^

GCA was first described by Gagne in 1969.^[1]^ As a rare disease process, GCA was mainly found in appendix, which can present with acute appendicitis, chronic abdominal pain, or no symptoms at all.^[17]^ Primary extra-appendiceal GCA, such as stomach, duodenum, ampulla of Vater, jejunum, ileum, cecum, splenic flexure and ascending colon, was extremely rare.^[1,3–6]^ To date, only 10 cases was reported about the GCA of rectum through literature review (Table 3). Our study gave a detailed account of an extremely rare case of anal canal GCA, which mainly in the anal canal but focally invading the lower rectum. Meanwhile, it was accompanied by perianal Paget disease. To date, no similar case was reported. From Table 3, we found the median age at diagnosis of GCA in rectum or anal canal is 64 years. Most of them showed good prognosis by surgical operation with adjuvant chemotherapy. However, a few of them showed recurrence or remote metastasis, which resulting in poor prognosis.

**Table 3:** Reported cases of goblet cell adenocarcinoma in rectum or anal canal.

No.	Author	Yr	Age	M/F	Location	Coexisting component	Tumor stage	Sychronous metastasis	Treatment	Adjuvant chemotherapy	Site of recurrence	Treatment of recurrence	Prognosis (mo)
1	Hernandez^[7]^	1974	71	F	Rectum	n.d.	T2	None	Transanal resection	None	None	None	Alive
2	Hernandez^[7]^	1974	60	F	Rectum	n.d.	n.d.	None	Only biopsy	None	None	None	Alive
3	Yagihashi^[8]^	1999	55	M	Rectum	n.d.	T1	Lymph node	Low anterior resection	None	None	None	Alive
4	Ishii^[9]^	2001	71	M	Rectum	Typical carcinoids WDA	T4b	Regional lymph node	Mile operation	None	Sychronous site	None	Died(9)
5	Kato^[10]^	2002	44	M	Rectum	none	T3	None	Total colectomy (accompanied by UC)	None	Liver, brain, peritoneum	Right lobectomy of liver intraperitoneal administration Of CDDP	Died(39)
6	Wakahara^[11]^	2010	58	M	Rectum	Typical carcinoids	T4b	Pelvic lymph node	Total pelvic exenteration, LLND	5-Fu, LV	Groin and pelvic lymph node	Lymphadenectomy	Alive(60)
7	Yamabuki^[12]^	2011	75	M	Rectum	None	T2	Regional lymph node	Mile operation	None	Lung	FOLFOX	Alive(15)
8	Gui X^[4]^	2011	80	M	Rectum	None	n.d.	None	n.d.	None	None	n.d.	Lost follow-up
9	Kang^[13]^	2016	46	F	Rectum	None	T1	None	Low anterior resection	None	None	None	Alive(10)
10	Inoue Y^[14]^	2020	77	M	Rectum	WDA	T3	None	Mile operation	None	Liver	FOLFOX + BV, CapeOX + BV, IRIS + BV	Alive(27)
11	Present case	2022	74	F	Anal canal	Paget disease	T1	None	Mile operation	5-Fu,	None	None	Alive(24)

5-Fu = LV fluorouracil and leucovorin; oxaliplatin, BV = Bevacizumab, CapeOX = capecitabine + oxaliplatin, CDDP = cisplatin, LLND = later lymph node dissection, n.d. = not described, UC = uncreative colitis, WDA = well-differentiated adenocarcinoma.

GCA was previously known as goblet cell carcinoid (GCC) or adenocarcinoid, as it was characterized with mixed phenotype of dual neuroendocrine and glandular differentiation. Its clinical behavior could range from relatively indolent disease that are completely cured by excision to malignant disease that spread widely and even cause death.^[18,19]^ The appendicular GCA was more aggressive compared with typical well-differentiated neuroendocrine tumors of the appendix but less aggressive compared with adenocarcinomas. For this reason, goblet cell carcinoid has been regarded as a distinctive entity, and is classified and staged as appendiceal adenocarcinomas.^[17]^ In 2019 World Health Organization, GCA became the preferred terminology for this appendiceal tumor.^[20]^ In our present case, the tumor was composed of signet-ring-like cells on confluent growth model and copious mucin was produced as well. Meanwhile, the tumor cells were positive for Syn, CD56, CDX2, CEA, Villin, and CK 20, further confirming its dual neuroendocrine and glandular differentiation.

GCA had an incidence of approximately 0.12 cases per 1 million individuals per year.^[21,22]^ Appendiceal GCC was reported in approximately 14% to 19% of primary appendiceal cancers.^[23]^ The median age at diagnosis was 58 years and there was no significant difference between males and females.^[23]^ To date, no specific risk factors have been identified although rare cases of GCA have been associated with schistosomiasis.^[24]^ Our report found the anal canal GCA was accompanied by perianal Paget disease, hinting it was possible that Paget disease was related to GCA. So careful digital rectal examination was important in the patient with perianal Paget disease for avoid missing diagnosis of GCA on anal canal.

The characteristic pathological findings of GCA included goblet cell morphology with rich intracytoplasmic mucin and a small compressed nucleus, and concentric infiltration of the appendiceal wall by small tight clusters, cords, or nests of tumor cells.^[25]^ The 3 tier grading systems of GCA was based on the proportion of goblet-like mucinous cells. Presence of at least 75% low grade pattern (tubular/nested architecture) was required for qualification of a tumor as grade 1, while grade 3 tumors should contain >50% high grade pattern (complex cribriform sheet-like growth pattern and/or single mucinous or non-mucinous cells).^[20]^ In our case, confluent growth model or complex cribriform sheet-like growth pattern was showed, so it was grade 3 according to the grading systems. Focally, the vessel invasion was found in this case, revealed the possibility of the poor prognosis in grade 3.

The derivation of GCA was still unclear. Some report revealed the GCA was derived from undifferentiated stem cells, which was different from typical carcinoids that originate from Kulchitsky in the mucosal stroma.^[26]^ The 2019 WHO classification, primary anal adenocarcinoma was divided into 2 subtypes: mucosal origin (arising from the luminal mucosa) and extramucosal origin (arising from anal glands, an anal fistula or non-fistulating glandular structures).^[20]^ Peripheral to the normal anal glands, we found individual adenocarcinoma in situ, hinting GCA maybe originate from anal glands. However, large number of investigation are needed for confirming this hypothesis in the future.

## 4. Conclusions

In a conclusion, goblet cell tumors was rare and easily under-recognizable, especially occurred in extra-appendiceal lesion. Careful digital rectal examination was important in the patient with perianal Paget disease for avoid missing diagnosis of GCA on anal canal. Some of them may show aggressive clinical behavior. Therefore, we should pay more attention on the recognization of this rare disease.

## Author contributions

**Investigation:** Xiaofei Yao.

**Project administration:** Minhua Li.

**Writing – original draft:** Xiaofei Yao.

**Writing – review & editing:** Minhua Li.

## References

[1] Abdalla AS Khan KA Shah A. Colonic goblet cell carcinoid: rarity of a rarity! A case report and review of literature. Chirurgia (Bucur). 2020;115:102–11. 32155405 10.21614/chirurgia.115.1.102

[2] Wang Y Shahabi A Loeffler A. Appendiceal goblet cell adenocarcinoma: a historically informed reading of 6 cases. Arch Pathol Lab Med. 2022;146:1402–11. 35142802 10.5858/arpa.2021-0249-RA

[3] Shibuya H Hijioka S Mizuno N. A rare case of ampullary goblet cell carcinoid. Intern Med. 2018;57:2489–96. 29607953 10.2169/internalmedicine.0516-17 PMC6172535

[4] Gui X Qin L Gao ZH. Goblet cell carcinoids at extraappendiceal locations of gastrointestinal tract: an underrecognized diagnostic pitfall. J Surg Oncol. 2011;103:790–5. 21240989 10.1002/jso.21863

[5] Gupta A Patel T Dargar P. Metastatic appendiceal goblet cell carcinoid masquerading as mucinous adenocarcinoma in effusion cytology: a diagnostic pitfall. J Cytol. 2013;30:136–8. 23833405 10.4103/0970-9371.112659 PMC3701339

[6] Jang KY Park HS Noh SJ. Adenocarcinoma ex-goblet cell carcinoid of the ascending colon concurrent with conventional adenocarcinoma. Pathology (Phila). 2018;50:789–92. 10.1016/j.pathol.2018.06.005 30314646

[7] Hernandez FJ Fernandez BB. Mucus-secreting colonic carcinoid tumors: light- and electron-microscopic study of three cases. Dis Colon Rectum. 1974;17:387–96. 4133878 10.1007/BF02586991

[8] Yagihashi N Koyama M Yagihashi S. Rectal adenocarcinoid with lymph node metastasis. Pathol Int. 1999;49:563–5. 10469401 10.1046/j.1440-1827.1999.00904.x

[9] Ishii Y Wakaki K Ishizawa S. Goblet cell carcinoid of the rectum—a case report. J Jpn Soc Clin Cytol. 2001;40:616–21.

[10] Kato H Hayashi K Sugihara K. A case report of ulcerative colitis accompanied by goblet cell carcinoid and adenocarcinoma. Nihon Shokakibyo Gakkai Zasshi. 2002;99:500–4. 12048894

[11] Wakahara T Yamamoto S Fujita S. A case of advanced rectal adenocarcinoid tumor with long-term survival. Jpn J Clin Oncol. 2010;40:690–3. 20338947 10.1093/jjco/hyq033

[12] Yamabuki T Omi M Yonemori A. Goblet cell carcinoid of the rectum with lymph node metastasis: report of a case. Surg Today. 2011;41:1284–9. 21874432 10.1007/s00595-010-4474-y

[13] Kang Y Choi JW Kim Y. Goblet cell carcinoid of the rectum in a patient with neurofibromatosis type 1. J Pathol Transl Med. 2016;50:482–5. 27237132 10.4132/jptm.2016.02.27 PMC5122723

[14] Inoue Y Horie H Homma Y. Goblet cell carcinoid of the rectum: a case report. Surg Case Rep. 2020;6:174. 32683504 10.1186/s40792-020-00937-3 PMC7368876

[15] Knoop KA Newberry RD. Goblet cells: multifaceted players in immunity at mucosal surfaces. Mucosal Immunol. 2018;11:1551–7. 29867079 10.1038/s41385-018-0039-y PMC8767637

[16] Hodges RR Bair JA Carozza RB. Signaling pathways used by EGF to stimulate conjunctival goblet cell secretion. Exp Eye Res. 2012;103:99–113. 22975404 10.1016/j.exer.2012.08.010 PMC3595052

[17] Sigley K Franklin M Welch S. Appendiceal goblet cell adenocarcinoma case report and review of the literature. Cureus. 2021;13:e13511. 33786220 10.7759/cureus.13511 PMC7992912

[18] Nagtegaal ID Odze RD Klimstra D. WHO Classification of Tumors Editorial Board. The 2019 WHO classification of tumors of the digestive system. Histopathology. 2020;76:182–8. 31433515 10.1111/his.13975 PMC7003895

[19] Rossi RE Luong TV Caplin ME. Goblet cell appendiceal tumors--management dilemmas and long-term outcomes. Surg Oncol. 2015;24:47–53. 25686643 10.1016/j.suronc.2015.01.001

[20] Ahadi M Sokolova A Brown I. 2019 World Health Organization Classification of appendiceal, colorectal and anal canal tumors: an update and critical assessment. Pathology (Phila). 2021;53:454–61. 10.1016/j.pathol.2020.10.010 33461799

[21] McCusker ME Coté TR Clegg LX. Primary malignant neoplasms of the appendix: a population-based study from the surveillance, epidemiology and end-results program, 1973-1998. Cancer. 2002;94:3307–12. 12115365 10.1002/cncr.10589

[22] McGory ML Maggard MA Kang H. Malignancies of the appendix: beyond case series reports. Dis Colon Rectum. 2005;48:2264–71. 16258711 10.1007/s10350-005-0196-4

[23] Gilmore G Jensen K Saligram S. Goblet cell carcinoid of the appendix - diagnostic challenges and treatment updates: a case report and review of the literature. J Med Case Rep. 2018;12:275. 30244681 10.1186/s13256-018-1789-6 PMC6151924

[24] Kelly KJ. Management of appendix cancer. Clin Colon Rectal Surg. 2015;28:247–55. 26648795 10.1055/s-0035-1564433 PMC4655112

[25] Zhang K Meyerson C Kassardjian A. Goblet cell carcinoid/carcinoma: an update. Adv Anat Pathol. 2019;26:75–83. 30601149 10.1097/PAP.0000000000000222

[26] Höfler H Klöppel G Heitz PU. Combined production of mucus, amines and peptides by goblet-cell carcinoids of the appendix and ileum. Pathol Res Pract. 1984;178:555–61. 6483683 10.1016/S0344-0338(84)80088-6

